# PI3Kγ inhibition drives M1 macrophage differentiation and synergizes with PD-L1 blockade to improve survival in poorly immunogenic head and neck squamous cell carcinoma

**DOI:** 10.1080/15384047.2025.2600701

**Published:** 2025-12-22

**Authors:** Pete P. Jordanides, Sushmitha Jagadeesha, Puja Upadhaya, Nathan M. Ryan, Kelvin Anderson, Felipe F. Lamenza, Suvekshya Shrestha, Arham Siddiqui, Anna R. Bopp, Sherefuddin H. Pracha, Peyton Roth, Reegan Kehres, Xiaokui Mo, Steve Oghumu

**Affiliations:** a Department of Pathology, Pelotonia Institute for Immuno-Oncology, The Ohio State University Comprehensive Cancer Center, College of Medicine, The Ohio State University Wexner Medical Center, Columbus, OH, USA; b Department of Biomedical Informatics, Center for Biostatistics, Pelotonia Institute of Immuno-Oncology, The Ohio State University, Wexner Medical Center, Columbus, OH, USA

**Keywords:** Head and neck squamous cell carcinoma, immunotherapy, PI3Kγ inhibition, PD-L1 checkpoint blockade, tumor microenvironment

## Abstract

**Background:**

Head and neck squamous cell carcinoma (HNSCC) is the sixth most common cancer globally with high mortality rates, highlighting the urgent need for novel therapeutic strategies. We investigated the efficacy of combining phosphoinositide 3-kinase gamma (PI3Kγ) inhibition with programmed death-ligand 1 (PD-L1) blockade in a poorly immunogenic HNSCC model.

**Materials and methods:**

Mouse bone marrow-derived macrophages (BMDMs) were differentiated and polarized in the presence or absence of the PI3Kγ inhibitor IPI-549 or culture supernatants from MOC2 cells treated with or without IPI-549. MOC2 cells were orthotopically injected into C57BL/6 mice, and treated with anti-PD-L1, IPI-549, combined anti-PD-L1 and IPI-549 or vehicle control. Tumor burden, survival, and immunological responses were evaluated.

**Results and conclusion:**

Dual inhibition of PI3Kγ (using IPI-549) and PD-L1 demonstrated nearly significant reduction in primary tumor burden and significantly increased survival compared to single or control treatments. PI3Kγ inhibition promoted macrophage differentiation toward an antitumoral M1 phenotype. In the bone marrow, dual therapy significantly increased MHC-II expression across various myeloid cell subsets and effectively normalized myelopoiesis. Notably, combination therapy increased CD8+ T-cell infiltration into tumors while decreasing T-cell exhaustion marker (LAG-3, CTLA-4, and TIM-3) and protumoral cytokine (IL-4). Combined PI3Kγ and PD-L1 inhibition offers a promising strategy for treating poorly immunogenic HNSCC by simultaneously targeting multiple immunosuppressive mechanisms. These findings provide a strong rationale for combining PI3Kγ and PD-L1 inhibitors as a therapeutic strategy for poorly immunogenic HNSCC, potentially improving clinical outcomes for patients.

## Introduction

Immunotherapy has emerged as one of the most promising strategies in the treatment of various cancers, including head and neck squamous cell carcinoma (HNSCC).^1^ In contrast to conventional treatments such as surgery, chemotherapy, and radiation, which directly targets the tumor, immunotherapy harnesses the body’s immune system to identify and destroy tumor cells.^2^ In HNSCC, particularly among Human Papilloma Virus (HPV)-negative cases, immunotherapy has become a critical area of focus owing to limitations of standard treatment regimens.^3^ These tumors frequently exhibit resistance to chemotherapy and radiation, and surgical interventions are associated with substantial physical and psychological morbidity.^4^ Despite being the sixth most common cancer worldwide and associated with high mortality, HNSCC treatment options have led to minimal improvements in patient outcomes. Five-year survival rates remain stagnant at approximately 40%–50%, emphasizing the urgent need for more effective approaches to improve patient outcomes.^5^ Immunotherapy presents a potential breakthrough by targeting specific molecular pathways that regulate immune responses, such as programmed cell death (PD-1)/programmed cell death ligand 1 (PD-L1) checkpoints, to increase survival.^6^

The PD-1/PD-L1 immune checkpoint signaling pathway promotes tumor resistance to immune-induced cell death, and immunotherapeutic approaches that target this axis effectively block tumor immune evasion. Anti-PD1/PD-L1 treatments have achieved remarkable clinical success in disrupting the ability of cancer cells to escape immune surveillance over the past decade.^7^ In HNSCC, PD-L1 has become a key therapeutic target, with many HPV-negative patients overexpressing PD-1 as high as 46%–87%.^8‐10^ However, when anti-PD-L1 checkpoint inhibitors have been used in clinical testing, suboptimal response rates have been observed. A Phase I clinical investigation with a PD-L1 inhibitor (atezolizumab) demonstrated an optimal response rate of 22% in HNSCC patients. This is comparable to the response rates seen with other PD-L1 inhibitors.^11^ Anti-PD-1 therapies alone provide durable responses only in a limited subset of patients, which underscores the need for novel therapeutic strategies, such as dual pathway targeting, to overcome resistance mechanisms.^3^ The concurrent inhibition of cytotoxic T-lymphocyte associated protein 4 (CTLA-4) and PD-L1 has demonstrated a synergistic effect in metastatic melanoma, enhancing efficacy Compared to mono therapeutic approaches.^10^ Similar combinatorial immunotherapeutic strategies can be exploited as a potential treatment paradigm for HNSCC.

Phosphoinositide 3-kinase (PI3K) is a catalytic enzyme that is part of the PI3K-AKT-mTOR pathway, which regulates cell proliferation, survival, and motility. In the context of HNSCC, PI3K plays a pivotal role, as one of the most mutated pathways, with a frequency of 66% in clinical cases.^12^ Furthermore, tumors with alterations in the PI3K pathway have higher incidence of mutations than tumors without a PI3K pathway alteration.^5^ These mutations highlight the importance of PI3K signaling in HNSCC, which provides a possible target for the modulation of HNSCC immunotherapy. Class 1B PI3K, or PI3Kγ, is an isoform of PI3K that promotes an immunosuppressive phenotype by modulating the tumor immune microenvironment. PI3Kγ inhibition has been shown in the past to increase the antitumor potential of T-cells and macrophages.^13‐15^ This inhibition has been shown to help overcome the resistance of checkpoint inhibitors, paving the way for a Phase I clinical trial targeting PI3Kγ (NCT02637531) in patients with solid tumors.^16^ We previously showed that genetic ablation of PI3Kγ improves antitumoral responses against HNSCC. Mice with a genetic deletion of PI3Kγ injected with an HNSCC cell line were found to have increased CD8+ T-cell infiltration and granzyme B production in the tumor microenvironment.^17^ However, despite these positive findings, tumor growth was minimally affected, and these mice showed increased expression of PD-L1 in multiple cell types in both the tumoral and lymph node microenvironments, potentially counteracting the favorable immune responses observed in tumor-bearing Pik3cgs^−/−^ mice.^17^ This finding suggests a tumor immune evasion mechanism through adaptive resistance, such that blockade of an immunosuppressive pathway (PI3K-*γ*) amplifies another immunosuppressive mechanism (PD-L1 expression) in HNSCC tumor cells, ultimately maintaining immune escape. These results provide a rationale for investigating the efficacy of combination therapy targeting both PI3K-*γ* and PD-L1 in HNSCC treatment.

Based on these observations from prior research, we investigated the effects of PI3Kγ inhibition using the small-molecule inhibitor IPI-549 (Eganelisib) against HNSCC. IPI-549 is a potent, first-in-class, highly selective PI3K-*γ* inhibitor that is orally bioavailable and possesses an excellent pharmacokinetic profile.^18^ We determined the efficacy of combined IPI-549 with a PD-L1 checkpoint inhibitor on HNSCC tumor outcomes and myeloid cell differentiation in vitro and in vivo. Our preclinical in vivo HNSCC model involved orthotopic implantation of Mouse Oral Carcinoma 2 (MOC2) HNSCC cells into the oral mucosa of C57BL/6 immunocompetent mice. These MOC2 cells display genetic alterations and dysregulated signaling pathways that are commonly seen in HNSCC, underscoring the model's translational relevance.^19^^,^^20^ Dual inhibition of PI3Kγ and PD-L1 resulted in enhanced survival outcomes in our HNSCC murine model. These findings suggest that targeting the PI3Kγ and PD-L1 pathways concurrently could represent a promising therapeutic approach for improving immunotherapy outcomes in HNSCC, potentially overcoming the immunosuppressive barriers that limit current treatment strategies.

## Results

### PI3Kγ inhibition promotes macrophage differentiation and M1 polarization while upregulating PD-L1 expression

**Figure 1.**
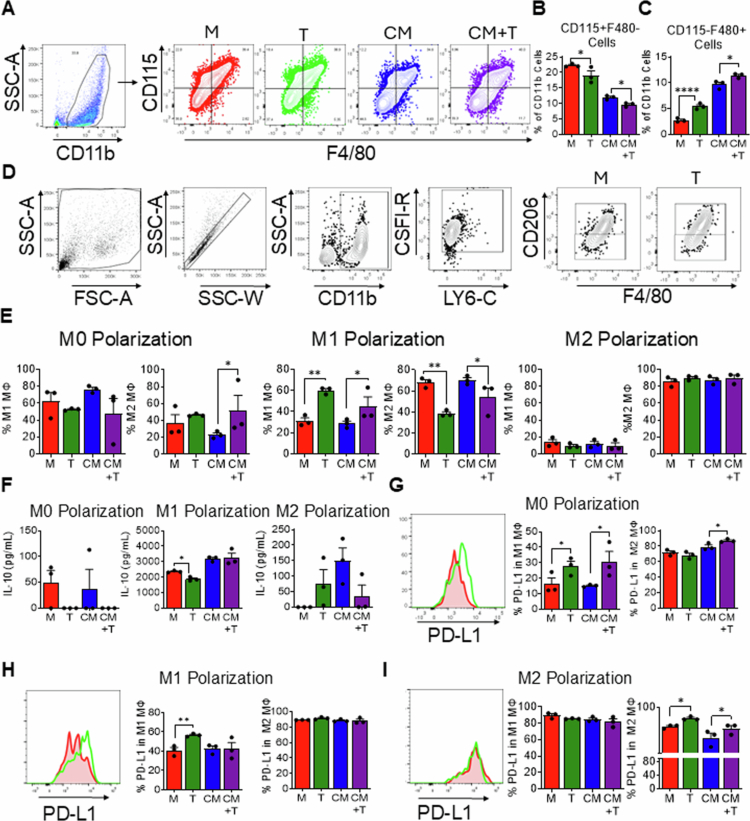
PI3Kγ inhibition promotes macrophage differentiation and M1 polarization while upregulating PD-L1 expression. (A) Representative flow cytometric plots illustrating macrophage differentiation across four different conditions: no treatment (M), IPI-549 treatment (T), conditioned media (CM) derived from HNSCC supernatants, and conditioned media treated with IPI-549 (CM + T). (B-C) Bar graphs showing the average percentages of (B) CD115+F4/80− cells and (C) CD115−F4/80+ cells from an *in vitro* macrophage differentiation experiment. (D) Representative flow cytometric plots showing the gating strategy used to distinguish the M1 and M2 macrophage subsets following *in vitro* macrophage polarization experiment. (E) Bar graphs showing the percentage of M1, M2 macrophage population across different macrophage polarization conditions. (F) Bar graphs quantifying IL-10 (pg/mL) production measured by ELISA across different macrophage polarization conditions. (G–I) PD-L1 expression in M1 and M2 macrophages from the different treatment groups acrossM0, M1 and M2 polarized conditions respectively. The data are presented as mean ± SD. *p-value < 0.1; **p-value < 0.01; and ****p-value < 0.0001, after multiple comparison adjustment.

Previous studies from our laboratory demonstrated that genetic PI3Kγ deletion in a murine HNSCC model resulted in enhanced antitumor responses mediated by increased T-cell cytotoxicity. Concurrently, we observed the upregulation of immunosuppressive markers in myeloid cells, which attenuated the therapeutic efficacy of PI3K inhibition^17^. We determined whether PI3K inhibitor-mediated alterations in myeloid cell function in HNSCC were attributable to direct effects on myeloid cells or indirect mechanisms mediated through cancer cell-derived factors representative of the tumor microenvironment. BMDMs were treated with IPI-549 or with supernatants from MOC2 HNSCC cells exposed to IPI-549, and we determined the effects on macrophage differentiation (Figure 1A). Treatment of BMDMs with IPI-549 resulted in a significant decrease in CD115+F4/80− cells (monocytes) and a significant increase in CD115−F4/80+ cells (macrophages) compared to non-IPI-549 treated controls (Figure 1B and C). Our results revealed that PI3K inhibition promotes myeloid cell differentiation into mature macrophages through direct and indirect mechanisms. Macrophages are a major subset of myeloid immune cells that play an important role in HNSCC tumor immunity. Classically activated macrophages (M1) promote proinflammatory and antitumoral effect, while alternatively activated macrophages (M2) promote antiinflammatory and protumoral effects^20^. To determine whether PI3Kγ inhibition favors macrophage polarization toward an antitumor or protumor phenotype, we examined the effect of IPI-549 cells on BMDMs following different macrophage polarization conditions (Figure 1D). No significant differences were observed in the M1 or M2 populations under M0 (nonpolarizing) conditions, except for a notable increase in M2 macrophages in the HNSCC culture supernatants treated with IPI-549 group compared to untreated conditioned media. In M1-polarized cultures, IPI-549 treatment resulted in a significant increase in M1 macrophages and a decrease in M2 macrophages. Conditioned media did not affect M1 or M2 macrophage polarization compared to control media (Figure 1E). No significant changes were observed in the M2-polarized cultures (Figure 1E). These results indicate that PI3K inhibition by IPI-549 directly promotes M1 polarization and can act on HNSCC tumor cells to promote M1 polarization, which potentially enhances antitumor immune responses.

Analysis of cytokine production from BMDM supernatants further supported the antitumor effect of PI3K inhibition, with a significant decrease in IL-10 levels in M1 macrophages directly treated with IPI-549 (Figure 1F). Given our previous *in vivo* observations of increased PD-L1 expression in myeloid cells from HNSCC tumor-bearing PI3K knockout mice, we examined PD-L1 expression in the presence or absence of IPI-549 across macrophage phenotypes and polarizing conditions. In fully differentiated macrophages cultured without M1 or M2 polarization, treatment with IPI-549 increased PD-L1 expression in M1 macrophages (Figure 1G). Direct treatment with IPI-549, but not supernatants from IPI-549 treated HNSCC cells, upregulated PD-L1 expression in M1 macrophages under M1 polarizing conditions (Figure 1H). Under M2 polarizing conditions, treatment with IPI-549 upregulated PD-L1 expression in M2 macrophages (Figure 1I). Our results demonstrate that while PI3Kγ inhibition by IPI-549 potentially contributes to an antitumor response by promoting M1 macrophage polarization, it was insufficient to suppress PD-L1 expression in myeloid cells.

### Dual PI3Kγ/PD-L1 inhibition mitigates tumor progression and inhibits PD-L1 expression in M1 macrophages in an orthotopic murine model of HNSCC

**Figure 2.**
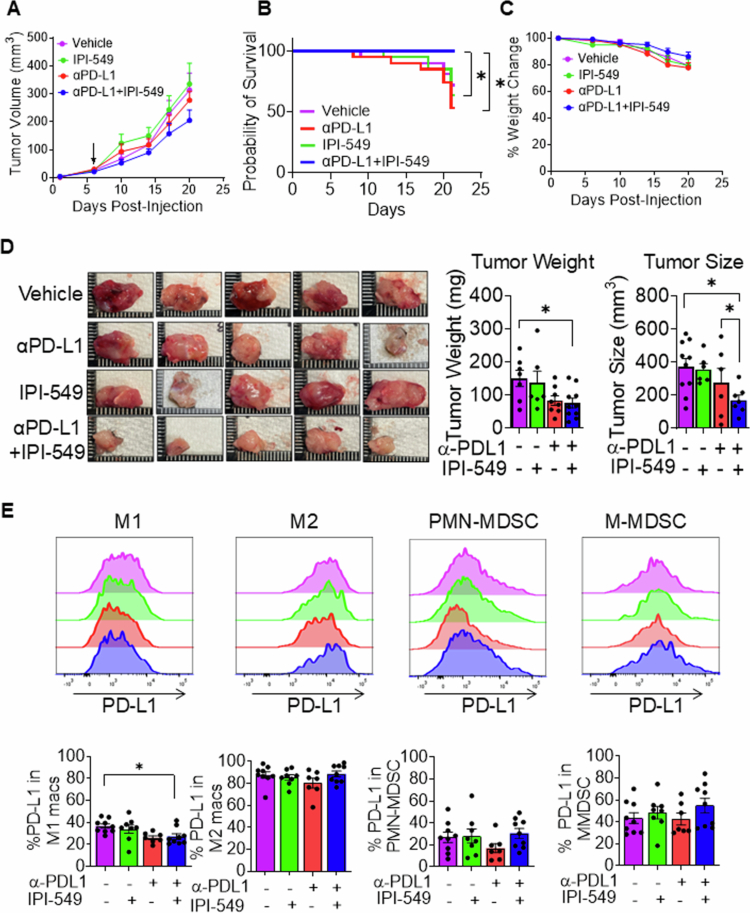
Dual PI3Kγ/PD-L1 inhibition mitigates tumor progression and inhibits PD-L1 expression in M1 macrophages in an orthotopic murine model of HNSCC. (A) Tumor volume (mm^3^) measurements of MOC2 HNSCC-injected mice treated with IPI-549, anti-PD-L1, anti-PD-L1 + IPI-549, or vehicle control. (B) Kaplan‒Meier survival analysis of tumor-bearing mice treated with IPI-549, anti-PD-L1, anti-PD-L1 + IPI-549, or vehicle control. (C) Bar graphs showing the percent change in mice weights (g) treated with IPI-549, anti-PD-L1, anti-PD-L1 + IPI-549, or vehicle control through the course of the experiment (D) Representative images of HNSCC tumors, along with a bar graph showing tumor weights and sizes of tumors harvested from each treatment group. (E) Histogram plots showing PD-L1 expression on M1, M2 macrophages, G-MDSC, and M-MDSC in tumors of mice treated with IPI-549, anti-PD-L1, and anti-PD-L1 + IPI-549, or vehicle control, as analyzed by flow cytometry. Bar graphs showing average PD-L1 expression in each group are also shown. The data are presented as mean ± SE. *p-value < 0.1 and **p-value < 0.01.

Based on these in vitro findings and our previous research, we hypothesized that combining PD-L1 blockade with PI3K inhibition could counteract the immunosuppressive effects seen with PI3K inhibition alone, potentially leading to enhanced therapeutic outcomes. To elucidate the therapeutic potential of dual PI3Kγ/PD-L1 inhibition in HNSCC, we orthotopically injected MOC2 cells into the right buccal mucosa of immunocompetent C57BL/6 mice and treated them with anti-PD-L1, IPI-549, anti-PD-L1 + IPI-549, or vehicle control. Consistent with the previously established kinetics of this highly aggressive HNSCC cell line, tumor progression reached the predetermined ethical endpoint criteria by day 21 post injection. Tumor volumetric analysis revealed that while all the treatment groups exhibited characteristic tumor growth patterns, the mice receiving dual treatment demonstrated slightly lower but not significant tumor volumes (Figure 2A). Assessment of animal survival across treatment groups revealed significant differences, with the dual treatment group exhibiting 100% survival at the experimental endpoint compared to single treatment and control mouse groups, where 50%–70% mortality was observed (Figure 2B). No significant differences in mouse weight were observed between single and dual treatment groups, suggesting the potential absence of overt toxicity (Figure 2C). Notably, primary tumors were visibly smallest in size among tumor-bearing mice that were treated with PI3Kγ/PD-L1 PI3K compared to single treatment and vehicle control groups (Figure 2D). Moreover, primary tumor weight analysis indicated a significant reduction in tumor weight in the dual treatment group compared to the vehicle control group. ImageJ-based quantification of tumor size revealed a significant reduction in the dual treatment group compared to both the vehicle and anti-PD-L1 treatment groups. (Figure 2D) Altogether our phenotypic analysis of a preclinical model of highly aggressive, poorly immunogenic HNSCC tumors provide evidence that targeting PI3Kγ and PD-L1 signaling pathways could potentially enhance therapeutic efficacy against HNSCC. Next, we quantified PD-L1 expression within distinct myeloid cell populations isolated from MOC-2 tumors of anti-PD-L1, IPI-549, anti-PD-L1 + IPI-549, or vehicle control-treated mice^21^ (Supplementary Figure 1). Notably, the dual treatment group exhibited significant reduction in PD-L1 levels in M1 macrophages. The diminished PD-L1 expression observed in the dual treatment corroborates our *in vivo* phenotypic findings demonstrating enhanced antitumor efficacy with combination therapy. No significant alterations in PD-L1 expression were detected across treatment groups within M2 macrophages or MDSCs (Figure 2E). Collectively, these findings demonstrate that dual PI3K/PD-L1 inhibition moderately inhibits tumor development in a highly aggressive experimental murine HNSCC model, which is in part associated with reduced PD-L1 expression on M1 macrophages.

### Reduced HNSCC tumor development by dual IPI-549/anti-PD-L1 treatment is associated with inhibition of MDSC-mediated immunosuppression

**Figure 3.**
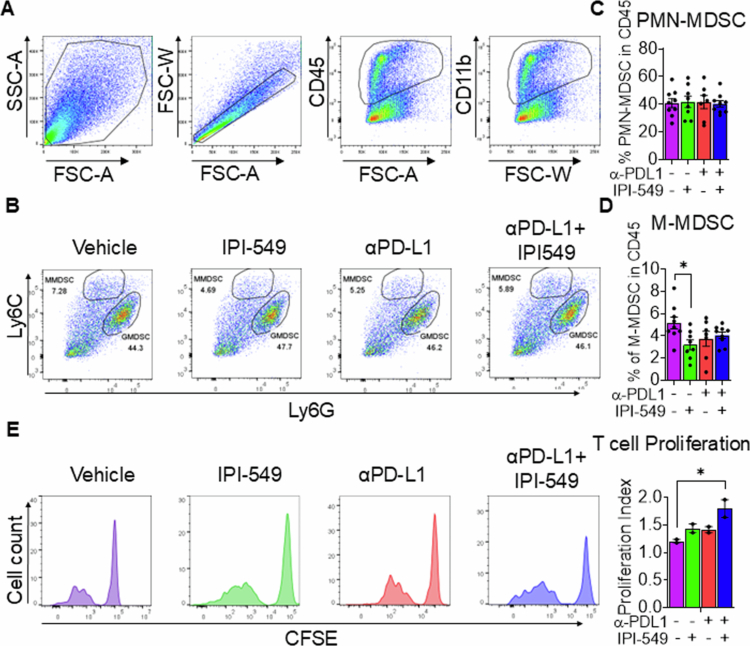
Reduced HNSCC tumor development by dual IPI-549/anti-PD-L1 treatment is associated with the inhibition of MDSC-mediated immunosuppression. (A-B) Representative flow cytometric dot plots showing the gating strategy for CD11b, G-MDSC, and M-MDSC from tumors of mice treated with IPI-549, anti-PD-L1, anti-PD-L1 + IPI-549, or vehicle control (C-D) Bar graph showing the percentages of (C) G-MDSC and (D) M-MDSC in the tumors of the mice in the different treatment groups. (E) Flow cytometric histograms depicting T-cell proliferation from MDSC: T-cell coculture across the different treatment groups. MDSCs were isolated from tumor-bearing mice treated with IPI-549, anti-PD-L1, anti-PD-L1 + IPI-549, or vehicle control. Bar graphs showing the proliferation indices of T-cells are also shown. The data are represented as mean ± SE. *p-value < 0.1.

To determine the potential underlying mechanisms associated with HNSCC inhibition by dual IPI-549/anti-PD-L1 treatment, we investigated its impact on myeloid cell recruitment and activity. Based on our previous work on the effects of PI3K and PD-L1 signaling on immunosuppressive myeloid cells, we first examined whether dual PI3K/PD-L1 inhibition differentially altered the infiltration of PMN-MDSCs and M-MDSCs into the tumor microenvironment (Figure 3A and B). Flow cytometric analysis revealed no significant changes in PMN-MDSC infiltration among any treatment groups (Figure 3C). Interestingly, we observed a reduction in the M-MDSC population following IPI-549 inhibitor alone treatment (Figure 3D). Next, we determined the impact of PI3K and/or PD-L1 inhibition on the immunosuppressive activity of MDSCs. To do this, isolated MDSCs from the tumors of MOC2 HNSCC tumor-bearing mice that had been treated with anti-PD-L1, IPI-549, anti-PD-L1 + IPI-549, or vehicle control were co-cultured with CD3/CD28 activated CFSE labeled T-cells isolated from C57BL/6 mice, and T-cell proliferation was measured by flow cytometry. Our analysis revealed that the greatest increase in proliferating T-cells was those that were cocultured with MDSCs treated with dual IPI-549/anti-PD-L1 (Figure 3E). These results demonstrate that dual targeting of the PI3Kγ and PD-L1 pathways inhibit MDSC mediated immunosuppressive functions in the HNSCC tumor microenvironment.

### Dual PI3Kγ/PD-L1 treatment enhances antigen presentation capacity in myeloid cell subtypes within tumor and spleen

**Figure 4.**
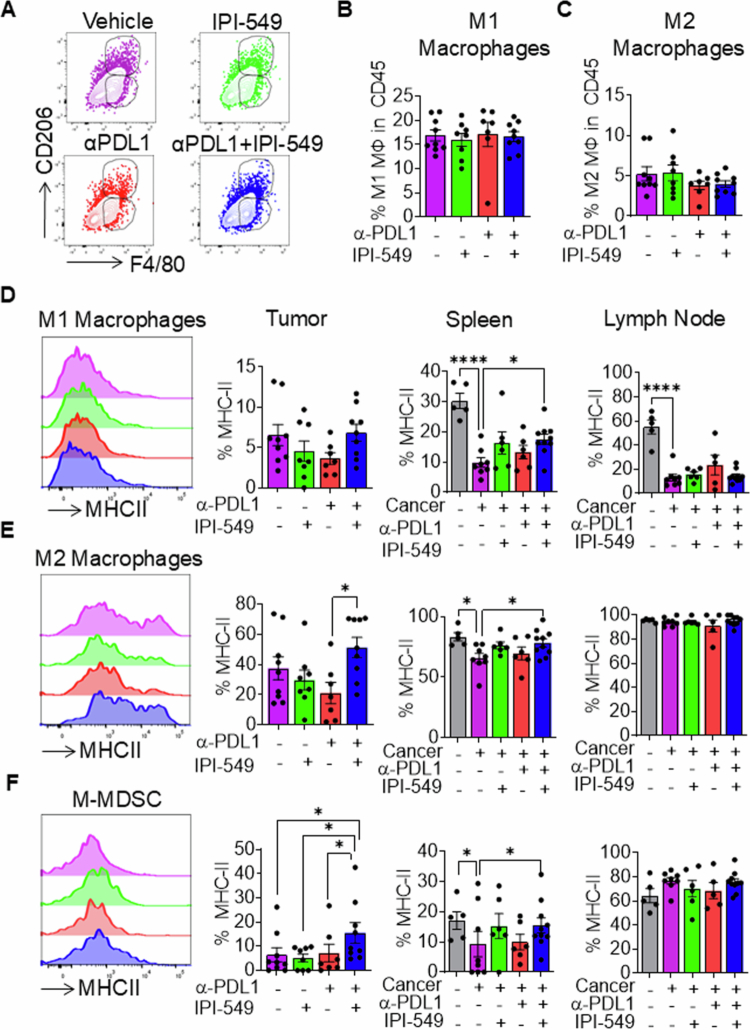
Dual PI3Kγ/PD-L1 treatment enhances antigen presentation capacity in myeloid cell subtypes within tumor and the spleen. (A) Representative flow cytometric plots showing M1 and M2 macrophage populations in tumors of HNSCC tumor-bearing mice treated with IPI-549, anti-PD-L1, anti-PD-L1 + IPI-549, or vehicle control. (B-C) Graph showing the percentage of (B) M1 and (C) M2 macrophages among CD45+ cells in tumors from different treatment groups. (D-F) Representative flow cytometry histogram plots and bar graphs showing the average percentage of MHC-II expression in (D) M1, (E) M2, and (F) M-MDSCs, in the tumor, spleen, and lymph nodes of sentinel and tumor-bearing mice treated with IPI-549, anti-PD-L1, anti-PD-L1 + IPI-549, or vehicle control. The data are represented as mean ± SE. *p-value < 0.1 and ****p-value < 0.0001.

To further determine the effects of dual PI3Kγ/PD-L1 inhibition on myeloid cells, which potentially contribute to reduced tumor growth in HNSCC tumor-bearing mice, we examined how macrophage phenotypes may impact the immune response to tumors following dual PI3Kγ/PD-L1 treatment. We assessed the distribution of M1 and M2 macrophage populations within the tumor microenvironment *in vivo*. We found no significant differences in the frequency of M1 or M2 macrophage subtypes across any of the treatment groups in these tumors (Figure 4A–C). We further examined MHC class II expression among various macrophage populations within the tumor, spleen, and tumor-draining lymph nodes. Our results showed an increase in MHCII expression in M2 macrophages within the tumor microenvironment of mice treated with dual PI3Kγ/PD-L1 inhibitors, suggestive of enhanced antigen presenting capabilities of tumor-associated macrophages are critical for optimal antitumor responses (Figure 4D–E). In the spleen of in dual PI3Kγ/PD-L1 treatment of tumor-bearing mice, the expression of MHCII in M1 and M2 macrophages was restored to its pretumoral state. However, no changes in MHCII expression were observed in the lymph nodes (Figure 4D–E). In M-MDSCs of the tumor microenvironment, expression of MHCII was enhanced following dual PI3Kγ/PD-L1 treatment of tumor-bearing mice compared to single treatment and vehicle control groups. No differences were observed in this population of myeloid cells in the draining lymph nodes (Figure 4F). Together, the increase in MHC II expression in myeloid cell subsets systemically and in the tumor microenvironment of HNSCC tumor-bearing mice suggests that dual PI3Kγ/PD-L1 inhibition promotes increased antigen presentation and immune activation in poorly immunogenic HNSCC.

### Combined treatment of tumor-bearing mice with PI3Kγ and PD-L1 inhibitors promotes differentiation of bone marrow cells into mature phenotypes

**Figure 5.**
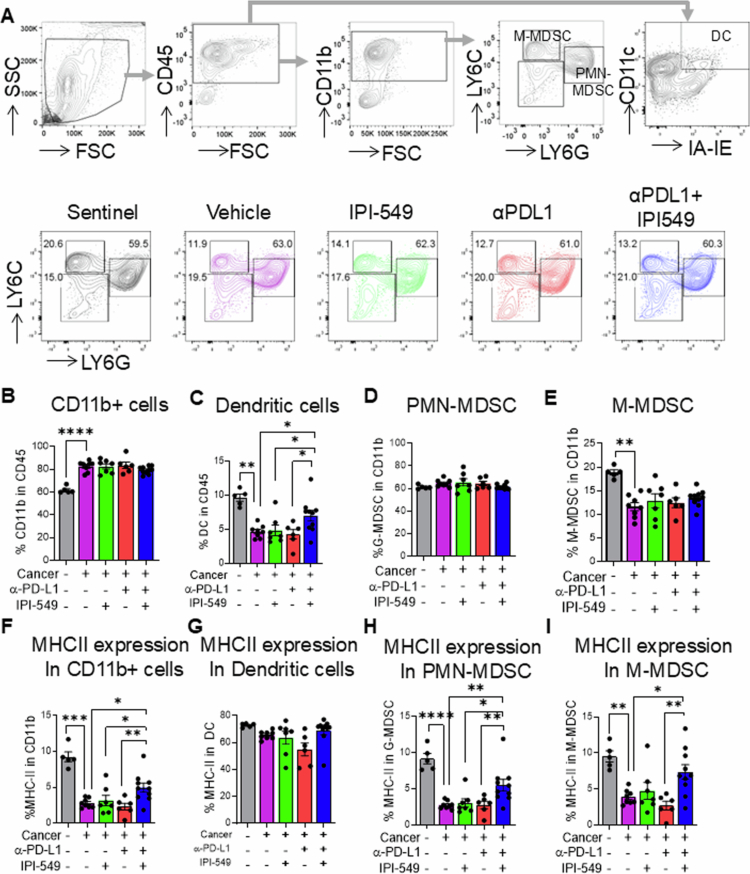
Combined treatment of tumor-bearing mice with PI3Kγ and PD-L1 inhibitors promotes the differentiation of bone marrow cells into mature phenotypes. (A) Representative flow cytometric plots showing the gating strategy for myeloid cell populations in the bone marrow of sentinel and HNSCC tumor-bearing mice treated with IPI-549, anti-PD-L1, anti-PD-L1 + PI-549, or vehicle control. Representative plots showing M-MDSC and PMN-MDSC populations for the different mouse treatment groups are also shown (B-E) Bar graph depicting the percentages of CD11b+ cells, dendritic cells, PMN-MDSCs and M-MDScs respectively in the bone marrow of different mouse groups (F-I) Bar graphs showing MHC-II expression in CD11b+ cells, dendritic cells, PMN-MDSCs and M-MDSC respectively in the bone marrow of different mouse groups. The data are represented as mean ± SE. *p-value < 0.1; **p-value < 0.01; ***p-value < 0.001; and ***p-value < 0.0001.

Given the systemic effects of PI3Kγ and PD-L1 inhibition during experimental HNSCC, we next determined the impact of this novel combination on myelopoiesis and terminal differentiation of specific myeloid subsets crucial for immunity against HNSCC within the bone marrow. Specifically, we analyzed total myeloid cells, dendritic cells, polymorphonuclear myeloid-derived suppressor cells (PMN-MDSC) and M-MDSC subsets (Figure 5A) and compared them against non-tumor-bearing control mice. As expected, myelopoiesis was significantly increased in tumor-bearing mice relative to non-tumor-bearing controls, and this increase was maintained in all treatment groups (Figure 5B). Among the myeloid subsets in the bone marrow, dual IPI-549/PD-L1 treatment significantly increased the proportion of dendritic cells in tumor-bearing mice relative to single treatment and vehicle controls and was comparable to the proportions in nontumor-bearing control mice (Figure 5C). The proportions of PMN-MDSCs did not differ across any of the treatment groups or the sentinel group. (Figure 5D). of M-MDSCs remained similar among treated and untreated tumor-bearing mice, which were significantly lower than those in nontumor-bearing mice (Figure 5E). Notably, among most of the myeloid cells tested, the combined IPI-549/PD-L1 treatment promoted significantly increased expression of MHC II compared to single and vehicle control treatment groups and almost restored MHC II levels to the levels observed in nontumor-bearing mice (Figure 5F–I). Interestingly, MHC II expression on PMN-MDSCs and M-MDSCs, which is typically characterized by low MHC II expression and immunosuppressive properties, was significantly increased by dual IPI-549/PD-L1 treatment (Figure 5H–I). This increased MHC II expression in myeloid cells in the bone marrow of tumor-bearing mice treated with a dual PI3Kγ/PD-L1 inhibitor suggests a systemic reprogramming of myeloid cells to promote terminal differentiation, immune activation and optimal priming of antitumoral T-cell responses against HNSCC.

### Dual PI3Kγ/PD-L1 inhibition enhances CD8+ T-cell infiltration and reduces T-cell exhaustion in the tumor microenvironment

**Figure 6.**
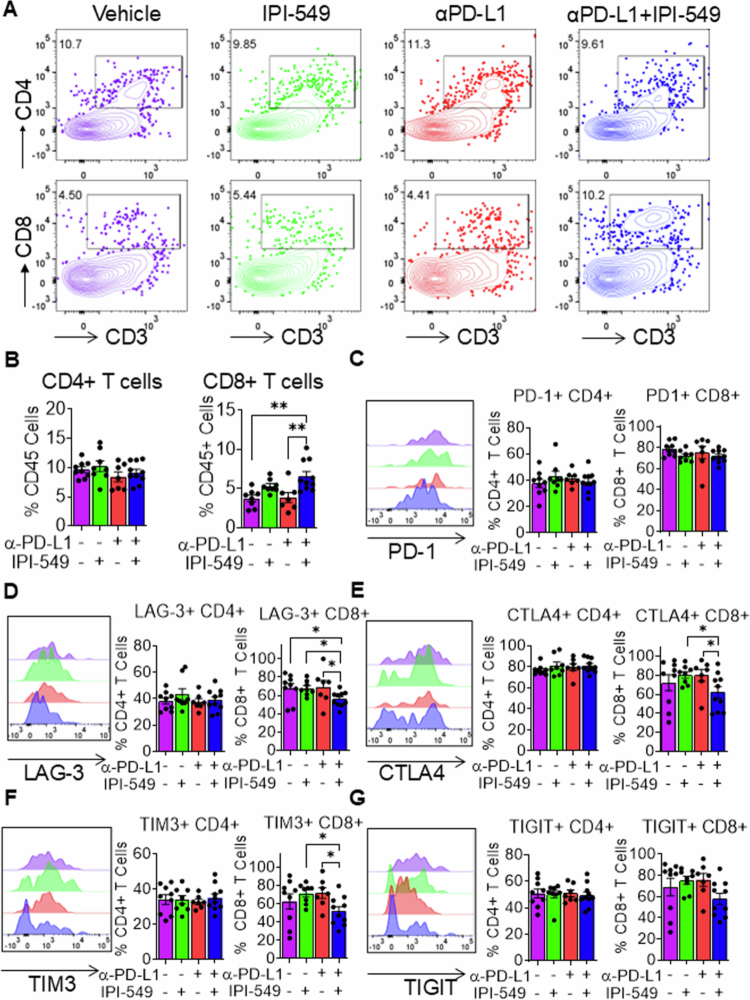
Dual PI3Kγ/PD-L1 inhibition enhances CD8+ T-cell infiltration and reduces T-cell exhaustion in the tumor microenvironment. (A). Flow cytometry plots of CD4^+^ and CD8+ T-cells in tumors of mice treated with IPI-549, anti-PD-L1, anti-PD-L1 + IPI-549, or vehicle control. (B) Bar graphs depicting percentages of CD4^+^ and CD8+ T-cells in the tumors of the different mouse treatment groups (C–G) Representative histogram plots and bar graphs depicting the percentage of (C) PD-1, (D) LAG-3, (E) CTLA-4, (F) TIM-3, (G) TIGIT in CD4^+^ and CD8+ T-cells in the tumors of HNSCC tumor-bearing mice treated with IPI-549, anti-PD-L1, anti-PD-L1 + IPI-549, or vehicle control. The data are presented as mean ± SE. *p-value < 0.1 and **p-value < 0.01.

Increased CD8+ T-cell infiltration into the HNSCC tumor microenvironment correlates with improved clinical outcomes and patient survival.^22^ Therefore, given the observed effects of dual PI3Kγ/PD-L1 inhibition on myeloid differentiation, activation and T-cell priming, we evaluated the impacts of this combined treatment regimen on T-cell infiltration and activation. First, we analyzed CD4^+^ and CD8+ T-cell infiltration into the tumor microenvironment (Figure 6A). Our analysis revealed significantly increased CD8+ T-cell infiltration in tumors from dual IPI-549/PD-L1 treated mice compared to PD-L1 or vehicle control treated mice (Figure 6B). This effect seemed to be driven by PI3Kγ inhibition, as IPI-549 single treatment also demonstrated increased CD8+ T-cell infiltration into the tumor microenvironment (Figure 6B), an effect that was also observed in our previous study using PI3Kγ knock out mice.^17^ CD4^+^ T-cell infiltration was not affected by PI3Kγ and/or anti-PD-L1 treatment. The enhanced CD8+ T-cell infiltration observed in IPI-549/PD-L1 treated mice suggests a stronger antitumor immune response, which is often correlated with better patient prognosis and improved survival which are supported by our phenotypic data. To determine the activation states of tumor-infiltrating T cells, we examined the expression of exhaustion markers on tumor-infiltrating CD4^+^ and CD8+ T cells. The overexpression of the coinhibitory receptors PD-1, CTLA-4, TIGIT, LAG-3, and TIM-3 drives T-cell dysfunction and exhaustion, thereby impairing their antitumor activity. Among tumor-infiltrating CD4^+^ and CD8+ T-cells, we observed no significant differences in PD-1^+^ expression in vehicle treated, dual IPI-549/PD-L1 treatment IPI-549/PD-L1-treated and single-treatment experimental groups (Figure 6C). LAG-3 expression in tumor-infiltrating CD8+ T-cells was significantly reduced in the IPI-549/PD-L1 dual treatment group compared to single and vehicle control treatment groups (Figure 6D). Additionally, dual PI3Kγ/PD-L1 treatment significantly reduced the expression of CTLA-4 and TIM-3 but not TIGIT in CD8+ T-cells compared to single treatment (Figure 6E–G). Reduction in mean fluorescence intensities (MFI) of LAG3, TIGIT and TIM3 in CD8+ tumor infiltrating lymphocytes of dual PI3Kγ/PD-L1 treatment was observed (Supplementary Figure 2). IPI-549 or PD-L1 single or dual treatments did not affect PD-1, CTLA-4, TIGIT, LAG-3, or TIM-3 expression in tumor-infiltrating CD4^+^ T-cells in HNSCC tumor-bearing mice (Figure 6C–G). These findings collectively demonstrate that inhibition of the PI3Kγ and PD-L1 signaling pathways enhances CD8+ T-cell tumor infiltration and reduces the expression of markers of CD8+ T-cell dysfunction in the tumor microenvironment.

### Effects of dual PI3Kγ/PD-L1 inhibitor therapy on CD8+ T-cell effector function against HNSCC

**Figure 7.**
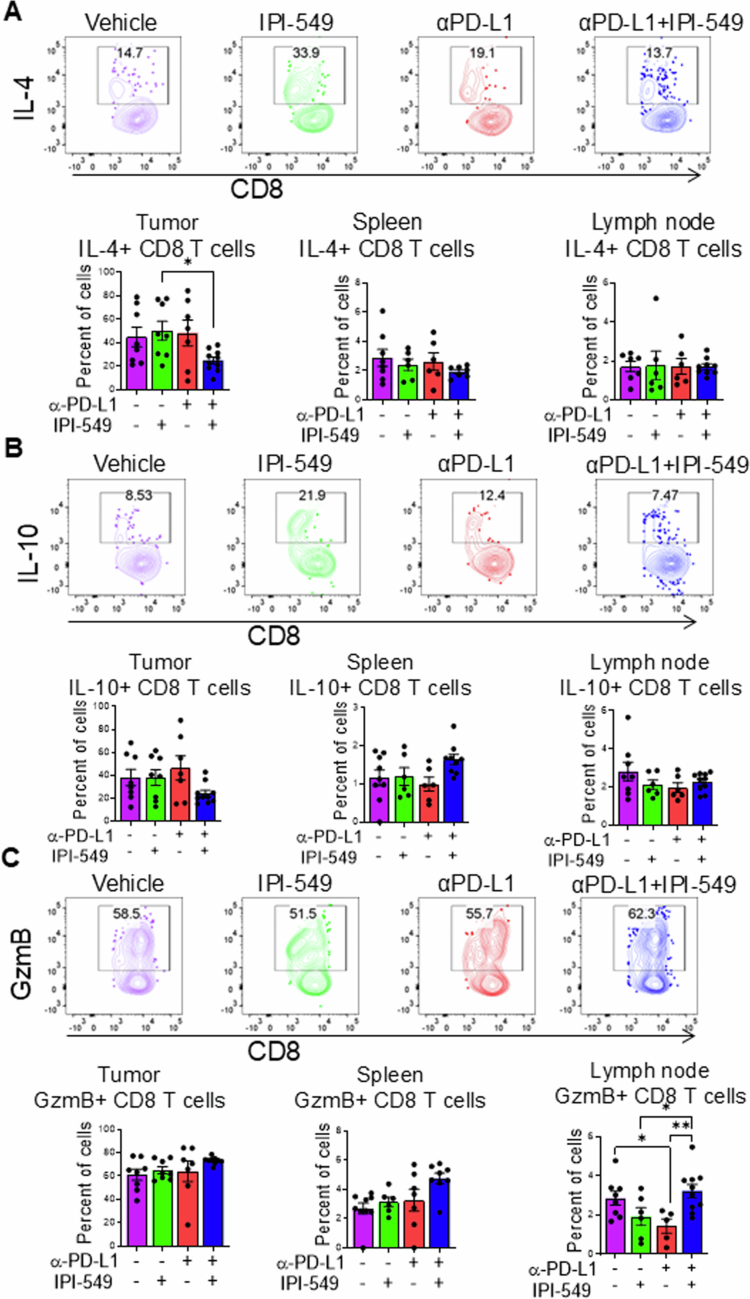
Effects of dual PI3Kγ/PD-L1 inhibitor therapy on CD8+ T-cell effector function against HNSCC. (A‒C) Representative flow cytometric plots and bar graphs depicting the percentage of the intracellular cytokines (A) IL-4, (B) IL-10, and (C) Granzyme B on CD8+ cells in the lymph node (LN), spleen (SP), and tumors (TM) of HNSCC tumor-bearing mice treated with IPI-549, anti-PD-L1, anti-PD-L1 + IPI-549, or vehicle control. The data are presented as mean ± SE. *p-value < 0.1 and **p-value < 0.01.

Given the observed increase in CD8+ T-cell infiltration at the primary tumor site and decreased expression of checkpoint inhibitor markers on CD8+ T-cells following dual IPI-549/PD-L1 treatment, we characterized the impact of this novel treatment on CD8+ T-cell function. First, we determined whether this combination approach altered the production of immunosuppressive cytokines that potentially counteract T-cell-mediated antitumor immunity. IL-4 and IL-10 suppress antitumor adaptive immunity and promote HNSCC tumor growth. Flow cytometric analysis revealed a significant reduction in the number of IL-4-expressing CD8+ T-cells in tumors from dual IPI-549/PD-L1-treated tumor-bearing mice compared to IPI-549 single-agent treatment groups (Figure 7A). No significant changes were observed for IL-10-producing CD8+ T-cells, among all the treatment groups (Figure 7B). Collectively, these data demonstrate that concurrent inhibition of PI3Kγ and PD-L1 slightly reduces the expression of immunosuppressive, protumorigenic cytokines within the tumor microenvironment, potentially contributing to enhanced therapeutic efficacy against HNSCC progression. Next, we investigated potential mechanisms associated with the antitumoral effects of CD8+ T-cells following dual IPI-549/PD-L1 therapy against HNSCC. We specifically determined the effects of PI3Kγ and PD-L1 inhibition on T-cell cytotoxic function and antitumor activity. We assessed granzyme B expression in CD8+ T-cells isolated from the tumors, tumor-draining lymph nodes, and spleens of MOC2 tumor-bearing mice across all treatment groups. Our results revealed elevated granzyme B expression in the lymph node CD8+ T-cells from dual IPI-549/PD-L1-treated tumor-bearing mice Compared to PD-L1 and IPI-549 single-treatment groups (Figure 7C). These results suggest moderately enhanced antitumoral activity from CD8+ T-cell populations in response to dual IPI-549/PD-L1 treatment.

## Discussion

Poorly immunogenic HNSCC remains therapeutically resistant, driven by multifaceted mechanisms that sustain immune evasion and limit durable response.^23^ Converging preclinical and clinical data underscore the rationale for developing combinatorial treatment strategies aimed at dismantling these barriers in otherwise unresponsive tumors. To experimentally model this challenge, we employed the MOC2 orthotopic model, a highly aggressive and metastatic murine HNSCC line that recapitulates the immune-excluded, treatment-resistant phenotype observed in patients. Our previous research on PI3Kγ inhibition highlighted the ability of HNSCC tumors to promote PD-L1 expression in the tumor microenvironment as a potential mechanism to evade the favorable immune response elicited by PI3Kγ inhibition.^17^ Given the suboptimal response rates to PD-L1 immunotherapy observed in clinical settings, we hypothesized that combining PI3Kγ inhibition with PD-L1 blockade might enhance therapeutic efficacy. This study investigated the efficacy and immunological mechanisms underlying this dual treatment approach against poorly immunogenic HNSCC.

In our MOC2 murine HNSCC cancer model, dual PI3Kγ/PD-L1 inhibition resulted in a reduced tumor burden and significantly improved survival compared with monotherapy, underscoring the therapeutic advantage of dual blockade. This observation aligns with findings from a previous HNSCC study employing dual PI3K/PD-L1 inhibition.^24^ However, we specifically selected the more aggressive MOC2 line, rather than the less aggressive MOC1 line used in that study, to test whether the combination would remain effective in a clinically relevant, high-risk context and to explore its mechanistic basis under these conditions. Furthermore, our study focuses on the specific inhibition of PI3Kγ versus the inhibition of both PI3Kγ and PI3Kδ (IPI-145) used in a prior study.^24^ It should be noted that IPI-145 and IPI-549 have different binding affinities for PI3Kγ, and unlike PI3Kγ, PI3Kδ (targeted only by IPI-145) is also involved in B cell activation. While our study employed a murine model, it offers valuable insights that could guide future research and clinical trials of this drug combination in HNSCC patients. Notably, a phase I clinical trial using the PI3Kγ inhibitor used in our current study (IPI-549) in combination with anti-PD-1 checkpoint blockade therapy in solid tumor patients was demonstrated to have safe and tolerable properties at 30–40 mg per day.^16^ A Phase II trial is currently ongoing. Our preclinical study provides a further rationale for, as well as underlying mechanisms associated with, HNSCC tumor control using this combinatorial treatment regimen.

In this study, we first assessed the impact of PI3Kγ inhibition on immune cell populations by exposing BMDMs to media conditioned by MOC2 HNSCC cells, as targeting the PI3Kγ pathway has been shown to reprogram macrophages in the tumor microenvironment to become less immunosuppressive. While previous studies have examined the effects of PI3Kγ inhibition on macrophages in vitro, our approach incorporated exposure to cancer-conditioned media to mimic the HNSCC tumor microenvironment.^14^ Our results demonstrated increased differentiation toward mature macrophage phenotypes when BMDMs were exposed to both direct IPI-549 treatment or the supernatants of IPI-549-treated MOC2 HNSCC cells compared to control groups. Furthermore, when macrophages underwent classical M1 activation, IPI-549 treatment increased the frequency of mature M1 macrophage populations and a concomitant reduction in M2 macrophage population. These results point to the efficacy of IPI-549-mediated PI3Kγ inhibition in macrophage reprogramming in the HNSCC tumor microenvironment via direct and indirect mechanisms. The recent MARIO-3 clinical trial investigating the combination therapy of PI3Kγ inhibition by Eganelisib, PD-L1 inhibition by atezolizumab, and nab-paclitaxel in patients with metastatic triple-negative breast cancers found a similar macrophage reprogramming from immunosuppressive tumor-associated macrophages to the M1 macrophage phenotype, as we have demonstrated^25^ Our *in vivo* experiments reflected a similar increase in the M1 macrophage population in tumor-bearing mice and a concurrent increase in PD-L1, which further strengthened our rationale for further studies in human patients presenting with immunologically inactive metastatic HNSCC.

Our dual treatment targeting PI3Kγ and PD-L1 was more effective than single treatment, as evidenced by a significant increase in MHC II expression in both M1 and M2 macrophages within the tumor microenvironment and spleen of HNSCC tumor-bearing mice following dual IPI-549/anti-PD-L1 treatment, effectively restoring these immune cells to an antitumoral phenotype. These observations suggest that antigen presentation capabilities are enhanced in the immunosuppressive HNSCC tumor microenvironment following dual PI3Kγ/PD-L1 inhibition, potentially overcoming the typically low immunogenicity of these tumors. MHC II molecules are crucial for antigen presentation of tumor-derived antigens to CD4^+^ ​​​​​T-cells, which are key to orchestrating broader adaptive antitumor immune responses. Previous research has shown that PI3Kγ inhibition can reprogram tumor-associated macrophages from an immunosuppressive M2 like state to a more proinflammatory M1-like phenotype.^14^ Our in vivo data demonstrate that MHC II expression increases across both macrophage populations, regardless of their initial polarization state, suggesting functional reprogramming toward improved antigen presentation. The combination of PI3Kγ inhibition and PD-L1 blockade appears to synergistically enhance this antigen-presenting capacity. While PI3Kγ inhibition likely drives the reprogramming of macrophages to an antigen-presenting phenotype, PD-L1 blockade may further enhance this effect by preventing the PD-1/PDL1-mediated suppression of MHC expression and antigen presentation.^26^

Macrophage differentiation and polarization, while critical to tumor progression, can be modulated by MDSCs. MDSCs are a diverse set of immature myeloid cell populations that have been shown to have a strong immunosuppressive effect on the T-cell immune response.^27^^,^^28^ The ability of MDSCs to augment the adaptive immune response and propagate tumor development has made them a target for cancer therapeutics.^29^ Inhibition of PI3Kγ has been shown to reduce MDSC-mediated suppression of T-cell responses and alter the tumor microenvironment in preclinical models.^15^ In tumor models, treatment with IPI-549 leads to a reduction in both the number and suppressive function of MDSCs, thereby boosting anti-tumor immune responses, especially when used alongside immune checkpoint inhibitors.^30^ While we did not observe significant differences in the MDSC populations in tumors of single and combined treated HNSCC tumor-bearing mice, functional analysis of MDSC populations in the tumor microenvironment of dual PI3Kγ/PD-L1 inhibitor treated experimental mice showed significant inhibition of MDSC mediated immunosuppression. This indicates that dual targeting of the PI3Kγ and PD-L1 pathways synergistically impacts MDSC function in a manner that contributes to the enhanced antitumoral immune response observed in treated mice.

Myeloid cell reprogramming by targeting the PI3Kγ pathway can enhance the cytotoxic T-cell antitumor response. However, there is evidence that demonstrates the possibility for T-cell suppression by inhibition of PI3Kγ and PI3Kδ.^31^ A recent *in vitro* study in the context of chronic lymphocytic leukemia shows enhanced T cell suppression by the inhibition of both PI3Kγ and PI3Kδ relative to the inhibition of just PI3Kγ alone and is posited to be a possible underlying cause for increase immune related side effects associated with the use of less specific inhibitors. It is therefore an important finding that a significant increase in CD8+ T-cell infiltration was observed in HNSCC tumors of mice receiving PI3Kγ and PD-L1 inhibition. This phenotype generally correlates with better prognosis and survival for HNSCC patients and likely contributed to the increased survival observed in this treatment group.^32^ Tumor-infiltrating CD8+ T-cells also expressed significantly lower levels of exhaustion markers, including LAG-3, CTLA4 and TIM3. LAG-3 is expressed on the surface of exhausted T-cells that inhibits CD8+ and CD4^+^ T-cell effector functions.^33^ LAG-3 inhibition has been shown in an immunocompetent mouse model to decrease tumor burden and increase the efficacy of CD8+ T-cell immune function.^34^^,^^35^ The decreased expression of these markers highlights the immunologic impact of dual treatment on CD8+ cytotoxic T-cells in the tumor microenvironment and their potentially enhanced capacity to kill HNSCC tumor cells.

Further examination of CD8+ T-cell effector function revealed decreased production of cytokines associated with tumor growth, such as IL-4, an immunomodulatory and inflammatory cytokine, which has been shown in vitro to facilitate growth and propagation of HNSCC.^36^ The decreased expression of IL-4 in the tumor microenvironment of IPI-549/PD-L1 treated tumor-bearing mice further emphasizes the anti-tumor effect of combined PI3Kγ and PD-L1 inhibition. Notably, these effects were more pronounced in the dual IPI-549/PD-L1 treatment group compared to single treatments, demonstrating the efficacy of this combinatorial approach. Further evidence of enhanced cytotoxicity in IPI-549/PD-L1 treated tumor-bearing mice is demonstrated by the increased production of the cytotoxic enzyme granzyme B in CD8+ T-cells of tumor draining lymph nodes.

Our study reveals that dual targeting of the PI3Kγ and PD-L1 pathways reduces the tumor burden and significantly improves survival in a highly aggressive, poorly immunogenic HNSCC mouse model. We demonstrate that this dual treatment approach elicits a favorable antitumor immune response in the tumor microenvironment, potentially mediated by myeloid cell reprogramming and enhanced cytotoxic T-cell infiltration and function. Our analysis further reveals the potential mechanisms underlying the modulation of myeloid immune cell populations in the tumor microenvironment mediated by PI3Kγ inhibition. Targeting PD-L1 in addition to PI3Kγ further inhibits MDSC mediated immunosuppression of antitumor T-cells, thereby promoting antitumoral responses against HNSCC. These findings support the combination of PI3Kγ inhibition with PD-L1 immunotherapy against HNSCC and suggest that this approach could improve clinical outcomes for patients with this challenging malignancy.

It should be noted that there are limitations to the current study, such as the use of a single cell line (MOC2), which limits the generalizability of the results. The use of additional HNSCC models to corroborate these findings and further characterize the therapeutic potential of dual PI3Kγ and PD-L1 pathway inhibition and associated immunological mechanisms will facilitate translational application of this treatment modality. Further, future studies should translate these findings to human HNSCC models, and ongoing clinical trials should validate the efficacy of this promising therapeutic strategy.

## Materials and methods

### Mice

Six- to eight-week-old female C57BL/6 mice were obtained from Jackson Laboratories (Bar Harbor, Maine, USA). For each experiment, the mice were randomly selected to receive oral orthotopic injections of MOC2 (*n* = 10 per group) or as sentinel controls (*n* = 5). Mouse numbers were selected based on prior studies with this model.^17,37‐40^ All animals were housed in an Ohio State University animal facility, following guidelines outlined by University Laboratory Animal Resources (ULAR). The mice were kept at a 12-h day‒night cycle and allowed food and water ad libitum. All the animal experiments were approved by the Institutional Animal Care and Use Committee (Approval number: 2018A00000054) and Institutional Biosafety Committee of the Ohio State University. The animals were euthanized using carbon dioxide gas, which was consistent with the recommendations of the American Veterinary Medical Association (AVMA) Guidelines for the Euthanasia of Animals. All experiments were reported in adherence to ARRIVE guidelines.

### Cell lines

The aggressive murine oral cancer cell line MOC2 was obtained from Kerafast (Boston, MA, USA). The cells were grown in IMDM/F12 media at a 2:1 ratio, along with 5% FBS, 1% pen-strep glutamine, 40 ng/mL hydrocortisone, 5 ng/mL human recombinant EGF, and 5 µg/mL insulin. The cells were grown to 75% confluence at 37 °C and 5% CO_2_ and harvested via trypsinization before being injected into the right buccal mucosa of the experimental mice.

### Orthotopic cancer model

A total of 3.0 * 10^4^ MOC2 cells suspended in ice-cold PBS were injected into the right buccal mucosa of C57BL/6 mice.^14^^,^^17^^,^^40^^,^^41^ Mouse weights and tumor sizes were measured biweekly using calipers, with tumor volumes estimated based on the formula V = (A × B²)/2 mm³, where A represents the longer tumor diameter and B represents the shorter diameter. The mice were sacrificed on the 21st day upon reaching early removal criteria (ERC). The tumors were allowed to grow for three weeks until terminal sacrifice, at which point the bone marrow, lymph nodes, spleen, and tumors were collected for further downstream analysis.

### Treatment regimen

Anti-PD-L1 monoclonal antibody and a rat IgG2b isotype control were purchased from Bio X Cell (Lebanon, NH, USA). The antibodies were administered intraperitoneally at a dose of 10 mg/kg per injection (0.5 mL at 0.5 mg/mL concentration) biweekly for a duration of two weeks, with treatment initiation on day 8 post-tumor inoculation. The PI3Kγ inhibitor IPI-549 was obtained from Chemietek (Indianapolis, IN, USA) and delivered via oral gavage at a dose of 15 mg/kg body weight in a vehicle consisting of 95% polyethylene glycol 400 and 5% N-methyl-2-pyrrolidone (PEG-400/NMP).^14^^,^^15^ Administration occurred daily for two weeks, beginning on day 8 post-tumor injection.

### Murine macrophage differentiation

MOC2 cells were cultured under two experimental conditions: (1) treated with 1 μM IPI-549 in 2 mL media and (2) the untreated control. Following 24 h of incubation, the cells were washed with PBS and cultured in 1 mL of media lacking hydrocortisone, human recombinant Epidermal Growth Factor (EGF), and insulin. Conditioned media from both conditions were subsequently collected. Bone marrow-derived macrophages (BMDMs) were isolated from the tibias of wild-type C57BL/6 mice and seeded at a density of 5 × 10^5^ cells/well. The BMDMs were allocated to four experimental groups: Group 1 contained the differentiation factor Colony Stimulating Factor-1 (CSF1) (M). Group 2 contained CSF1 and IPI-549 (T). Group 3 contained CSF1 with culture media from untreated MOC2 HNSCC cells (CM). Group 4 contained CSF1 with culture media from IPI-549 treated MOC2 HNSCC cells (CM+T). Differentiation factor CSF1 was added on day 0, and the respective MOC2 media conditions were introduced on day 3. The cells were harvested on day 7 post-treatment initiation. Flow cytometric analysis was performed on all the treatment groups, and both the cell supernatants and the cellular lysates were collected for subsequent analyses.

### Macrophage polarization

Following the 7-d BMDM differentiation period, BMDMs were subjected to macrophage polarization conditions across three separate culture plates: Non-polarized (M0) condition: BMDMs were cultured in standard culture media. M1 polarization condition: BMDMs were cultured in media supplemented with Interferon-gamma (IFN-*γ*) (20 ng/mL) and lipopolysaccharide (LPS) (100  ng/mL). M2 polarization condition: BMDMs were cultured in media supplemented with interleukin 4 (IL-4) (20 ng/mL). For each polarization condition, BMDMs were incubated with a polarizing cocktail alone (M), a polarizing cocktail + IPI-549 (T), a polarizing cocktail + media from untreated MOC2 cells (CM), polarizing cocktail + media from IPI-549 treated MOC2 cells (CM+T). All the plates were incubated for 24 h under standard culture conditions. Subsequently, flow cytometric analysis was performed to assess macrophage phenotype markers. The cell supernatants were harvested and preserved for downstream analysis.

### MDSC mediated T-cell proliferation assay

T-cells were first isolated from sentinel mice using the STEMCELL Technologies EasySep Mouse T-cell isolation Kit (catalog #19851). These isolated T-cells were subsequently stained with Carboxyfluorescein succinimidyl ester (CFSE) at a concentration of 1 µM. After washing, the labeled T-cells were transferred to 24-well plates that had been pre-coated with CD3/CD28. In parallel, Myeloid Derived Suppressor Cells (MDSCs) were isolated from both the tumor tissue and spleen of tumor-bearing mice across different treatment groups using the STEMCELL Technologies EasySep Mouse MDSC isolation Kit (Catalog # 19867). The isolated T-cells and MDSCs were then cocultured for 72 h, after which T cell proliferation was determined by flow cytometry.

### Flow cytometry

Single-cell suspensions were obtained from the lymph nodes, spleens, and tumors of the mice for flow cytometric analysis. Splenic cells were treated in ACK Lysis Buffer to lyse erythrocytes for 3 min. The cells isolated from the tumor tissue were digested in collagenase (Thermo Fisher Scientific; Waltham, MA) for 60 min at 37 °C and then passed through nylon mesh filters. 1 * 10^6^ cells were extracellularly stained with fluorochrome-conjugated antibodies for CD3, CD4, cytotoxic T-lymphocyte-associated protein-4 (CTLA-4), PD-1, CD69, CD45, lymphocyte activation gene 3 (LAG-3), CD49b, T-cell immunoreceptor with Ig and ITIM domains (TIGIT), T cell immunoreceptor with Ig and ITIM domains (TIM-3), and CD8. 2 * 10^6^ cells were isolated from these tissues and intracellularly stained with fluorochrome-conjugated antibodies for CD3, (Perforin (Prf), CD4, IL-4, IL-17, CD8, CD49b, Granzyme B (Gzmb), IL-10, and IFN-*γ*. For the myeloid extracellular panel, bone marrow single-cell suspensions were also analyzed. 1 * 10^6^ cells were extracellularly stained with fluorochrome-conjugated antibodies for IA/IE, Colony stimulating factor 1 receptor (CSF-1R), CD86, F4/80, CD11c, CD45, the lymphocyte antigen 6 complex, locus C1 (Ly6C), CD11b, Lymphocyte antigen 6 complex, locus G6D (Ly6G), PD-L1, and CD206. All the antibodies used in this experiment for flow cytometry were purchased from BioLegend (San Diego, CA, USA). Samples were run using a BD FACSCelesta flow cytometer (BD Biosciences), and subsequent analysis was performed using FlowJo software (Tree Star Inc., Ashland, OR, USA).

### ELISA

Supernatants collected from macrophage cultures were analyzed for the IL-10 cytokine profile using sandwich enzyme-linked immunosorbent assay (ELISA). Capture and detection antibodies were obtained from Biolegend (San Diego, CA, USA). Signal development was achieved using streptavidin-alkaline phosphatase (BD Biosciences, San Jose, CA, USA) followed by para-nitrophenyl phosphate substrate (Thermo-Fisher Scientific, Foster City, CA, USA) in glycine buffer. Absorbance measurements were acquired at 405 nm using a SpectraMax 190 microplate spectrophotometer (Molecular Devices; San Jose, CA, USA).

### Statistical analysis

Data were summarized using means and standard deviations. Experiments were performed twice. For the cell-based experiments, statistical analysis was performed using analysis of variance (ANOVA), with treatment as a fixed factor. In vivo tumor growth data were analyzed using a mixed-effects model, with treatment and time (days) as fixed effects and individual mice as a random effect to account for repeated measures. To meet the assumptions of normality, the data were log₂-transformed prior to analysis. When the log₂ transformation did not sufficiently normalize the residuals (e.g., in Figures 1E, 4E lymph node, and 7C), nonparametric Kruskal–Wallis tests were used. *P*-values were adjusted using the Hochberg step-up procedure to control the Type I error rate. All the statistical analyses were conducted using SAS software (SAS Institute, Cary, NC).

## Supplementary Material

Supplementary material Supplementary Figure 1.

Supplementary material Supplementary Figure 2.

## Data Availability

The data supporting the findings of this study can be obtained from the corresponding author (SO) upon request.
