# Supplementary figures and images for: PI3Kγ inhibition drives M1 macrophage differentiation and synergizes with PD-L1 blockade to improve survival in poorly immunogenic head and neck squamous cell carcinoma
Source: Cancer Biol Ther. 2025 Dec 22;27(1):2600701. doi: 10.1080/15384047.2025.2600701 (PMC12758254; doi:10.1080/15384047.2025.2600701)

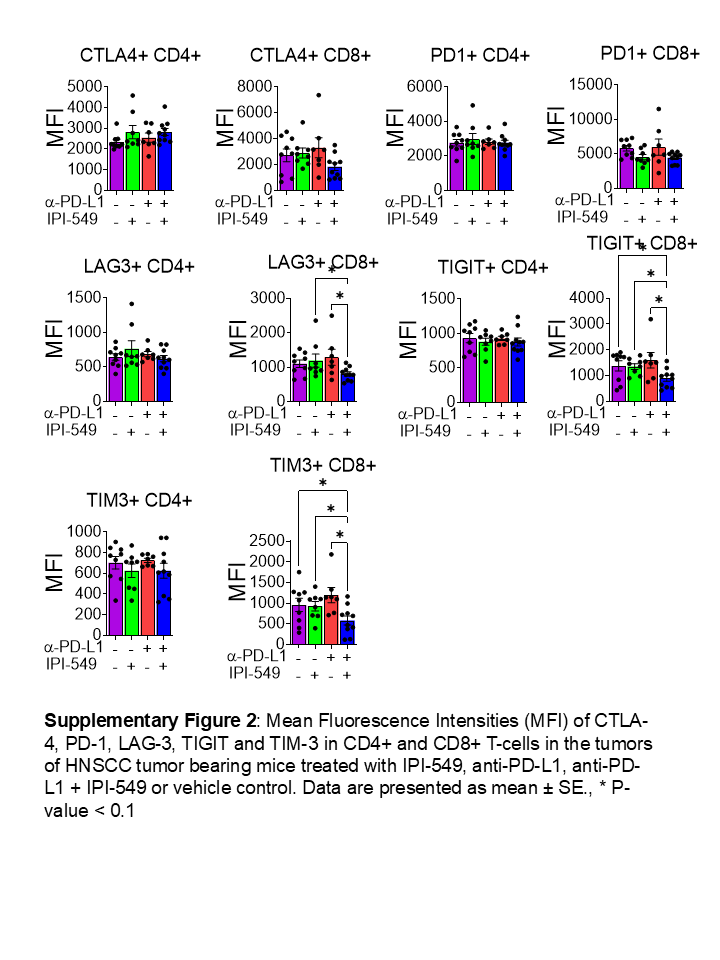

Supplement: Supplementary material — Supplementary Figure 1. [file KCBT_A_2600701_SM1629.tif]

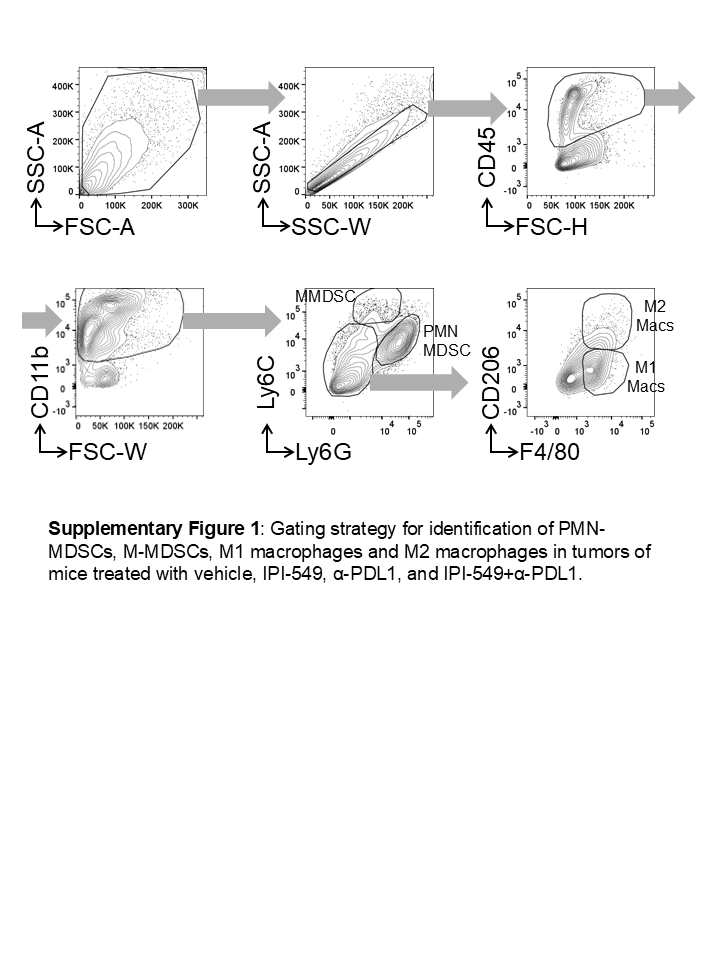

Supplement: Supplementary material — Supplementary Figure 2. [file KCBT_A_2600701_SM1626.tif]
